# Functional endurance capacity is associated with multiple other physical fitness components in 7–14-year-olds: a cross-sectional study

**DOI:** 10.1186/s12889-021-10702-2

**Published:** 2021-04-07

**Authors:** Mario Kasović, Lovro Štefan, Vilko Petrić, Vesna Štemberger, Iva Blažević

**Affiliations:** 1 Department of General and Applied Kinesiology, Faculty of Kinesiology, University of Zagreb, Horvaćanski zavoj 15, Zagreb, Croatia; 2 Department of Sport Motorics and Methodology in Kinanthropology, Faculty of Sports Studies, Masaryk University, Brno, Czech Republic; 3 Department of Educational Studies, Faculty of Teacher Education, University of Rijeka, Rijeka, Croatia; 4 Department of Primary Teacher Education, Faculty of Education, University of Ljubljana, Ljubljana, Slovenia; 5 Department of Primary Teacher Education, Faculty of Educational Sciences, University of Pula, Pula, Croatia

**Keywords:** Functional endurance capacity, Musculoskeletal fitness, School-aged children, Testing

## Abstract

**Background:**

Although evidence suggests that functional endurance capacity is the most important component associated with future health, little is known of how it is associated with multiple other physical fitness components. Since various physical fitness aspects do not change the same as functional endurance capacity during childhood, it is necessary to establish possible associations between functional endurance capacity and other physical fitness components in children. Therefore, the main purpose of the study was to test the associations between functional endurance capacity with other physical fitness components in 7–14-year-old children, stratified by gender.

**Methods:**

In this cross-sectional study, we recruited 1612 children [mean age ± standard deviation (SD) = 9.72 ± 2.37 years; 52.5% girls). Health-related physical fitness components included: 1) body-mass index (kg/m^2^) calculated from height and weight (measure of body size), 2) sit-and-reach test (measure of flexibility), 3) standing broad jump (measure of explosive strength of lower extremities), 4) sit-ups in 30 s (measure of repetitive strength of the trunk), 5) 10 × 5 shuttle run test (measure of agility) and 6) 20-m shuttle run test (measure of functional endurance capacity). The associations were performed using generalized estimating equations with beta (*β*) coefficients.

**Results:**

After adjusting for age, functional endurance capacity was associated with sit-and-reach test (*β* = 0.13, *p* < 0.001), standing broad jump (*β* = 0.59, *p* < 0.001), sit-ups in 30 s (*β* = 0.53, *p* < 0.001) and 10 × 5 shuttle run test (*β* = − 0.56, *p* < 0.001) in boys. In girls, functional endurance capacity was associated with body-mass index (*β* = − 0.12, *p* < 0.001), sit-and-reach test (*β* = 0.21, *p* < 0.001), standing broad jump (*β* = 0.25, *p* < 0.001), sit-ups in 30 s (*β* = 0.36, *p* < 0.001) and 10 × 5 shuttle run test (*β* = − 0.40, *p* < 0.001). No significant associations between functional endurance capacity and body-mass index in boys were observed.

**Conclusions:**

Although significant, functional endurance capacity is weakly to moderately associated with other physical fitness components, pointing out that such measure should be tested separately from other aspects of physical fitness in school-aged children.

## Background

Physical fitness has become an important non-communicable factor associated with well-being and health in the past two decades [1, 2]. It is often defined as ‘an integrated measure of most, if not all, the body functions (skeletomuscular, cardiorespiratory, hematocirculatory, psychoneurological and endocrine–metabolic) involved in the performance of daily physical activity and/or physical exercise’ [2]. Evidence suggests that higher levels of physical fitness in youth may prevent from cardiovascular, metabolic and mental diseases later in life [3–7], highlighting the importance of tracking characteristics of physical fitness from childhood to adulthood [8]. Moreover, studies have recognized that most risk factors attributed to chronic diseases in adulthood begin during childhood [2, 9, 10], pointing out that interventions aiming to enhance physical fitness at younger age for future health benefits are warranted [11].

Physical fitness represents a multifactorial construct and an integrated measure of body composition, functional endurance capacity, muscular fitness, speed/agility and flexibility [2]. Although all aspects of physical fitness seem to be important [2], functional endurance capacity has been the strongest predictor of morbidity and mortality in both men and women independently of other risk factors [12, 13]. In recent years, the level of functional endurance capacity has declined dramatically in school-aged children, increasing the prevalence of overweight/obesity [14] and not meeting the recommended levels of physical activity [15]. Of note, the ‘EUROFIT’, the’ ALPHA-FIT’ and the ‘FITNESS GRAM’ test batteries are the most widely applied in primary and secondary school students to assess the level of physical fitness.

Although functional endurance capacity has been associated with fundamental movement skill proficiency [16], a small proportion of studies have been provided regarding associations to other physical fitness components [11]. In general, evidence suggests that standing broad jump and agility shuttle run are the strongest predictors of functional endurance capacity (number of completed endurance shuttle run stages) with ≈10% of the variance shared in performance in these tests, followed by bent arm hang time, sit-ups (≈6–7%) and sit-and-reach test (≈3%) [11]. According to aforementioned, functional endurance capacity has been only weakly associated with multiple other aspects of physical fitness, concluding that this measure should be tested as a separate physical fitness component within the school system. Moreover, biological and environmental changes in functional endurance capacity do not follow the changes in other physical fitness components [11], i.e. the associations between functional endurance capacity and other aspects of physical fitness remain unclear. Specifically, a rather linear increase of absolute maximal oxygen uptake (VO_2max_) from childhood to adolescence has been observed previously, while speed has two separate growth spurts and muscle strength increases linearly during childhood, but has a remarkable growth spurt in boys during puberty and is more stable and reaches a plateau in girls [2].

Therefore, the main purpose of the study was to test the associations between functional endurance capacity with other physical fitness components in 7–14-year-old children, stratified by gender. We hypothesized, that functional endurance capacity would be positively associated with muscular and motor physical fitness, yet inversely associated with body size. If the associations happen to be weak, this will imply that functional endurance capacity is a single construct of overall physical fitness, which should be measured independently of multiple other physical fitness components.

## Methods

### Study participants

In this cross-sectional study, the participants were children aged 7–14 years from the city of Zagreb. At the first stage, a random sampling approach was used to select primary schools. Randomization of schools was done with replacement by drawing school codes on slips of paper from a box, with each school having equal probability of selection. At the second stage, we randomly selected one class presenting one age group within each school. Finally, 12 primary schools with 96 classes (12 schools × 8 classes) and 1950 students were selected. The inclusion criteria were: 1) being healthy without physical or mental problems diagnosed by the doctor, 2) regularly attending physical education classes and 3) those who had body weight and height measured and completed all physical fitness tests were included in the analysis. Of these, 338 did not have a measure of functional endurance capacity or were absent from school during the testing day. Analyses were performed on 1612 school aged children (response rate = 82.7, 52.5% girls). All procedures performed in this study were anonymous and were conducted according to Declaration of Helsinki. The study was approved by the Faculty of Kinesiology, University of Zagreb, Croatia. The informed consent voluntarily was signed by the participants, participants’ parents or their guardians.

### Functional endurance capacity

The 20-m shuttle run test was used to assess the level of functional endurance capacity [2–4, 11]. Detailed information about the testing procedure is described elsewhere [2–4, 11]. The final score was written as the number of stages completed during every-minute increasing pace of 20-m shuttle run test from walking to running. The result in 20-m shuttle run test provides a valid estimate of treadmill maximal oxygen uptake in youth [17].

### Multiple other physical fitness components

Other health-related physical fitness components included: 1) body-mass index (kg/m^2^) calculated from height and weight (measure of body size), 2) sit-and-reach test (measure of lower body flexibility), 3) standing broad jump (measure of explosive strength), 4) sit-ups in 30 s (measure of repetitive strength of the trunk) and 5) 10 × 5 shuttle run test (measure of agility). The same field – based tests have been used previously to assess the level of physical fitness in school – aged children [3, 11]. We followed the procedure of previously published study on the same topic [11, 18]. Height and weight were objectively measured by using stadiometer and digital scale with a precision of 0.1 cm and 01 kg. ***Body-mass index*** was calculated by dividing weight in kg with height in m^2^ [weight (kg)/height(m)^2^]. ***Sit-and-reach test*** was tested by trying to reach forward as far as possible keeping knees straight in a sitting position with feet vertical to the ground. ***Standing broad jump*** tests jumping distance from a standing start (‘frog leap‘). While performing the jumps, each child was asked to bend their knees with their arms in front of them, parallel to the ground, then to swing both arms, push off vigorously and jump forward as far as possible, trying to land with their feet together and stay upright [18]. ***Sit-up test*** evaluates repetitive strength of the trunk as number of sit-ups completed from lying position (knees bent at a 90°) in 30 s. Children were seated on the floor, backs straight, hands clasped behind their neck, knees bent at 90° with heels and feet flat on the mat. They then lay down on their backs, shoulders touching the mat, and returned to the sitting position with their elbows out in front to touch their knees, keeping the hands clasped behind their neck the whole time [18]. ***Agility shuttle run*** measures the time required to complete 50 m shuttle run test from a standing start during which the participants run forth and back five times to complete five 10 m laps [11].

According to previous studies, testing procedures performed in the study were standardized in order to minimize the effects of environmental factors and to avoid fatigue [11]. Before the study began, we had contacted principles from 16 schools to take part in the study. After the initial screening, 12 schools agreed to participate. Physical fitness was assessed from September to October and all school were evaluated at the same time. Prior the testing, each teacher was instructed about the testing methodology to standardize the procedure across all schools and classes. During the testing, children wore light T-shirt, shorts and training shoes. To avoid fatigue, tests were split into two non-consecutive days within 1 week [11]. On the first day of measurement, tests were administrated and performed by children in following order: 1) body-mass index, 2) sit-and-reach test, 3) standing broad jump, 4) sit-ups in 30 s and 5) 10 × 5 shuttle run test. On the second day, 20-m shuttle run test was performed [19]. The rest between each test was 5 min and between attempts within each test 3 min.

### Data analysis

Basic descriptive statistics of the study participants are presented as mean ± standard deviation. Kolmogorov-Smirnov test was applied to identify outliers, which were subsequently excluded. The associations between the functional endurance capacity (unadjusted model and model adjusted for age) with other multiple physical fitness components were determined by using generalized estimating equations. The working correlation matrix was set to exchangeable in all analyses. As highlighted in previous studies [20], the regression models were tested for several assumptions: 1) multicollinearity diagnostics using variance inflation index, 2) normality of residuals using the normal probability plot and histogram of residuals, and 3) heteroscedasticity using the standardized residuals vs. predicted plot. All assumptions were met for all regression models. Sex-specific analyses were performed, since there were significant differences between boys and girls in all physical fitness tests (*p* < 0.001; except for body-mass index, *p* = 0.210). Two-sided *p*-values were used, and significance was set at α < 0.05. All the analyses were calculated in Statistical Packages for Social Sciences v.23 (SPSS, Chicago, IL, United States).

## Results

Basic descriptive statistics of the study participants are presented in Table 1. Boys were taller, heavier and had higher body-mass index values. Boys performed better in all physical fitness tests, except for sit-and-reach test in favor to girls.

**Table 1:** Basic descriptive statistics of the study participants (*N* = 1612)

Study variables	Total sample (***N*** = 1612)	Boys (***N*** = 765)	Girls (***N =*** 847)	***p-***value*
	Mean (SD)	Mean (SD)	Mean (SD)	
**Age (years)**	9.7 (2.4)	9.8 (2.4)	9.6 (2.3)	0.148
**Height (cm)**	151.0 (176)	152.0 (19.4)	150.2 (15.7)	0.046
**Weight (kg)**	45.1 (19.1)	46.5 (13.3)	43.9 (14.04)	0.006
**Body-mass index (kg/m**^**2**^**)**	20.2 (3.4)	21.6 (3.6)	19.9 (3.3)	< 0.001
**Sit-and-reach test (cm)**	20.4 (8.3)	17.6 (8.3)	23.0 (7.3)	< 0.001
**Standing broad jump (cm)**	158.5 (43.5)	164.4 (38.9)	153.1 (46.7)	< 0.001
**Sit-ups in 30 s (reps)**	17.1 (7.0)	17.8 (7.2)	16.5 (6.8)	< 0.001
**10 × 5 shuttle run (sec)**	23.1 (3.0)	22.6 (3.2)	23.6 (2.8)	< 0.001
**20-m shuttle run (level)**	4.3 (1.9)	4.9 (2.2)	3.9 (1.4)	< 0.001

*denotes significant differences between boys and girls

*p* < 0.05

The associations between functional endurance capacity and multiple other physical fitness components in boys are presented in Table 2. In unadjusted model, variance of performance in standing broad jump (40.0%), 10 × 5 shuttle run (36.0%) and sit-ups in 30 s (32.5%) were each explained by functional endurance capacity the strongest, followed by weaker but still significant association by functional endurance capacity with sit-and-reach test (1.2%). When models were adjusted for age, variance of performance in standing broad jump (34.8%), 10 × 5 shuttle run (31.4%) and sit-ups in 30 s (28.1%) remained the strongest predictors of functional endurance capacity, followed by sit-and-reach test (1.7%). Body – mass index was not significantly associated with functional endurance capacity in both unadjusted and adjusted models.

**Table 2:** The associations between functional endurance capacity and multiple other physical fitness components in boys (*N* = 765)

Study variables	***β*** coefficient^a^	***t***-value	***p***-value
**Body-mass index (kg/m**^**2**^**)**			
Unadjusted model	0.01	0.118	0.906
Model adjusted by *age*	0.02	0.361	0.718
**Sit-and-reach test (cm)**			
Unadjusted model	0.11	2.897	0.004
Model adjusted by *age*	0.13	3.497	< 0.001
**Standing broad jump (cm)**			
Unadjusted model	0.63	21.127	< 0.001
Model adjusted by *age*	0.59	19.900	< 0.001
**Sit-ups in 30 s (reps)**			
Unadjusted model	0.57	18.332	< 0.001
Model adjusted by *age*	0.53	17.369	< 0.001
**10 × 5 shuttle run (sec)**			
Unadjusted model	−0.60	−19.739	< 0.001
Model adjusted by *age*	−0.56	−18.165	< 0.001

^a^standardized *β* coefficient

*p* < 0.05

The associations between functional endurance capacity and multiple other physical fitness components in girls are presented in Table 3. In unadjusted model, variance of performance in 10 × 5 shuttle run (22.1%) was the strongest predictor explained by functional endurance capacity, followed by sit-ups in 30 s (16.8%), standing broad jump (9.6%) and sit-and-reach test (7.3%). When models were adjusted for *age*, variance of performance in 10 × 5 shuttle run (16.3%), sit-ups in 30 s (13.0%) and standing broad jump (6.3%) remained the strongest predictors of functional endurance capacity, followed by sit-and-reach test (4.4%) and body-mass index (1.4%).

**Table 3:** The associations between functional endurance capacity and multiple other physical fitness components in girls (*N* = 857)

Study variables	***β*** coefficient^a^	***t***-value	***p***-value
**Body-mass index (kg/m**^**2**^**)**			
Unadjusted model	−0.05	−1.283	0.200
Model adjusted by *age*	−0.12	−3.597	< 0.001
**Sit-and-reach test (cm)**			
Unadjusted model	0.27	7.851	< 0.001
Model adjusted by *age*	0.21	6.528	< 0.001
**Standing broad jump (cm)**			
Unadjusted model	0.31	8.838	< 0.001
Model adjusted by *age*	0.25	7.698	< 0.001
**Sit-ups in 30 s (reps)**			
Unadjusted model	0.41	12.403	< 0.001
Model adjusted by *age*	0.36	11.457	< 0.001
**10 × 5 shuttle run (sec)**			
Unadjusted model	−0.47	−14.899	< 0.001
Model adjusted by *age*	−0.40	−12.531	< 0.001

^a^standardized *β* coefficient

*p* < 0.05

In the whole sample, variance of performance in standing broad jump run (35.1%) was the strongest predictor explained by functional endurance capacity, followed by 10 × 5 shuttle run (31.4%), sit-ups in 30 s (25.0%), sit-and-reach test (0.4%) and body – mass index (0.0%). When models were adjusted for *age*, variance of performance in standing broad jump (38.6%), 10 × 5 shuttle run (34.4%) and sit-ups in 30 s (31.1%) remained the strongest predictors of functional endurance capacity, followed by sit-and-reach test (11.5%) and body-mass index (11.5%).

## Discussion

The main purpose of the study was to test the associations between functional endurance capacity with other physical fitness components in 7–14-year-old children, stratified by gender. The main findings are: 1) functional endurance capacity predicts between 1 and 40% of the variance in performance in multiple other physical fitness components in boys, 2) in girls, functional endurance capacity predicts between 7 and 22% of the variance in performance in multiple other physical fitness components and 3) when adjusting for *age*, the percentage of variance shared between functional endurance capacity and multiple other physical fitness components slightly declines. Regarding gender differences in the associations between functional endurance capacity and other physical fitness components, girls not participating in sport tend to improve the level of functional endurance capacity much slower, compared to boys [1], while functional endurance capacity before puberty improves very slow in school – aged boys [21].

Our results of functional endurance capacity being most strongly associated to 10 × 5 shuttle run, standing broad jump and sit-ups in 30 s are in line with previous findings obtained among a large sample of Lithuanian school aged children [11]. Specifically, a study by Venckunas et al. [11] has shown that variance of performance in 10 × 5 shuttle run and standing broad jump were each explained by functional endurance capacity the strongest (> 10%), followed by the association between Functional endurance capacity with the abilities in bent arm hang and sit-ups (functional endurance capacity explaining ∼6.5% of the variance of the performance in these tests), as well as in balance and sit-and-reach tasks (functional endurance capacity significantly explaining ∼3% of the variance). It has been hypothesized, that for 10 × 5 shuttle run and standing broad jump performance, movement patterns are similar and the same muscle groups (i.e. leg extensors) need to be involved for locomotion [11]. Another potential mechanism may be the nature of these activities, which require different jumping, accelerating and decelerating performances deserving for intrinsic musculoskeletal characteristics, synchronizing upper and lower body and gaining appropriate momentum [11]. Also, the aforementioned tasks fall under weight-bearing exercises, which share similar moving patterns. Indeed, studies have shown that functional endurance capacity is associated with anaerobic functional capacities required for performing agility and power/strength tasks [20].

The strongest associations between functional endurance capacity and muscular fitness are not surprising, since higher levels of these components reduce the risk of all-cause mortality [22, 23] and are often interrelated [11]. From the perspective in sport, evidence suggests that low functional endurance capacity may be compensated for additional muscle training stimulus in aerobic endurance athletes [24], pointing out that all physical fitness components should be equally developed and enhanced across the lifespan. This supports the findings from previous studies, stating that being involved in endurance sport is not associated with an increased life expectancy [6]. Therefore, physical fitness, as a multifactorial construct, is the best non-communicable factor remotely associated to health [3–7]. Nevertheless, the critical period when physical fitness (especially functional endurance capacity) should be trained is during the childhood period, since it successfully predicts the development of cardiovascular diseases in later life [4].

This study has a few limitations. First, by using a cross-sectional design, we cannot determine the causality of the association, that is multiple other physical fitness components were associated to functional endurance capacity. Second, we randomly selected schools and classes for the purpose of this study and achieved an acceptable response rate. Nevertheless, more physically active families are more prone to participating in the studies of such nature [25]. Thus, potential selection bias cannot be excluded. Third, the proxy of functional endurance capacity was assessed through the 20-m shuttle run test. Although this test has been widely used and the reliability and validity properties have been confirmed [4], treadmill or bicycle ergometers may have given somewhat different maximal oxygen uptake values and associations between functional endurance capacity with multiple other physical fitness components. Studies have shown that the 20-m shuttle run test is designed to determine the maximal aerobic power [26], while more direct measurements assess VO_2max_. Thus, Fourth, body – mass index was used as a proxy of body composition. However, body – mass index cannot discriminate between fat mass and fat – free mass and more sophisticated tools, like dual X – ray absorptiometry must be used to assess the level of body composition in school – aged children. Finally, we did not assess the level of maturity. Although previous studies have recommended equations for maturity offset [27], standard errors of the equations are 0.53 years in girls and 0.54 years in boys. Also, a lack of fit between predicted and observed ages at peak height velocity (PHV) within each chronological age group and within each year before and after PHV has been observed previously [27]. Therefore, future research on the same topic needs to be longitudinal with more objective methods to assess the level of functional endurance capacity in school aged children.

## Conclusions

This study confirms that functional endurance capacity is most strongly associated with 10 × 5 shuttle run and standing broad jump, followed by sit-ups in 30 s, sit-and reach test and body-mass index. Although significant, the associations between functional endurance capacity and multiple other physical fitness components are weak to moderate, which is an important information of testing functional endurance capacity as a separate physical fitness component in school settings.

## Data Availability

The datasets used and/or analyzed during the current study are available from the corresponding author on reasonable request.

## Abbreviations

β: Beta
PHV: Peak height velocity
VO_2max_: Maximal oxygen uptake
